# Feasibility of Deep Learning-Based Segmentation of the Facial and Vestibulocochlear Nerves on High-Resolution Magnetic Resonance Imaging

**DOI:** 10.7759/cureus.108842

**Published:** 2026-05-14

**Authors:** Michael Bartellas, Yeshwant Chillakuru, Matthew Su, Sofiya Yusina, Daniel Jethanamest

**Affiliations:** 1 Otolaryngology - Head and Neck Surgery, NYU Langone, New York, USA

**Keywords:** cranial nerve segmentation, deep learning, facial nerve, mri, u-net, vestibulocochlear nerve

## Abstract

Objective: To evaluate the feasibility of deep learning-based automated segmentation of the facial and vestibulocochlear nerves within the cisternal and intracanalicular segments on high-resolution magnetic resonance imaging.

Study design and setting: This was a retrospective imaging study conducted at a tertiary referral center.

Patients: Twenty-two adult patients with normal internal auditory canal magnetic resonance imaging and no skull base pathology were included.

Methods: Manual segmentation of the facial and vestibulocochlear nerves was performed on axial constructive interference in steady state magnetic resonance images using 3D Slicer. The dataset was divided into training, validation, and test subsets. A three-dimensional U-Net convolutional neural network was trained with standard augmentation. Additional Medical Open Network for Artificial Intelligence-based architectures, including Attention U-Net, Dynamic U-Net, and U-Net++, were trained and compared under identical preprocessing and training conditions.

Results: All models generated anatomically plausible segmentations on qualitative review. The baseline U-Net achieved a test Dice similarity coefficient of 0.6398. Dynamic U-Net demonstrated the highest validation performance (Dice = 0.6236), while U-Net++ achieved the highest test performance (Dice = 0.6716). Attention U-Net demonstrated lower performance in this small-structure segmentation task. Performance trends were consistent with the challenges inherent to segmenting thin neural structures with limited voxel representation.

Conclusions: Deep learning-based segmentation of the facial and vestibulocochlear nerves on high-resolution magnetic resonance imaging is feasible within a limited retrospective dataset. Model selection appears important for small-structure segmentation, with Dynamic U-Net and U-Net++ demonstrating relatively higher performance trends within this limited dataset. Although performance metrics were modest and derived from a limited test dataset, automated segmentation showed consistent anatomic overlap with manual labels on qualitative review. These findings provide preliminary technical groundwork for future validation in larger cohorts and extension to clinically relevant applications such as cochlear nerve integrity in implant candidates.

## Introduction

The facial nerve (cranial nerve (CN) VII) and vestibulocochlear nerve (CN VIII) are small-caliber neural structures critical for hearing, balance, and facial movement. Accurate delineation of these nerves is important in the evaluation of vestibular schwannomas, assessment of cochlear nerve integrity in implant candidates, and surgical planning for lateral skull base procedures. Although these nerves are typically visualized on high-resolution magnetic resonance imaging (MRI), reliable segmentation can be challenging because of their slender morphology, close proximity, and limited contrast relative to surrounding cerebrospinal fluid. Manual segmentation remains the reference standard but is labor-intensive, time-consuming, and subject to interobserver variability.

Recent advances in deep learning have transformed medical image segmentation, particularly through convolutional neural network architectures such as U-Net and its derivatives [1-4]. These models have demonstrated strong performance across a range of medical imaging applications, including brain, spine, and inner ear segmentation [5-10]. However, segmentation performance typically declines for very small structures with limited voxel representation.

Prior studies have explored automated segmentation of small neural and vascular structures, including the trigeminal nerve and adjacent vasculature [5], facial nerve segmentation on temporal bone computed tomography (CT) [6], and multimodal cranial nerve tract segmentation [7]. While these efforts demonstrate the potential of deep learning for fine neural anatomy, application to the individual facial and vestibulocochlear nerves on high-resolution MRI remains limited.

Architectural modifications of the original U-Net, including attention mechanisms, nested skip connections, and dynamically configured encoder-decoder pathways, may influence performance in small-structure segmentation tasks. Frameworks such as the Medical Open Network for Artificial Intelligence (MONAI) enable standardized comparison of these architectures. However, the relative performance of these models for segmentation of very small cranial nerves in limited datasets remains unclear.

The present study aimed to evaluate the feasibility of deep learning-based automated segmentation of the facial and vestibulocochlear nerves within the cisternal and intracanalicular segments using high-resolution constructive interference in steady state (CISS) MRI sequences. By assessing quantitative segmentation performance and qualitative anatomic overlap, we sought to establish preliminary technical groundwork for future validation in larger and pathologic cohorts.

This article was previously presented as a poster at the American Neurotology Society Spring Meeting on April 24, 2026.

## Materials and methods

Study design and cohort

**Figure 1:**
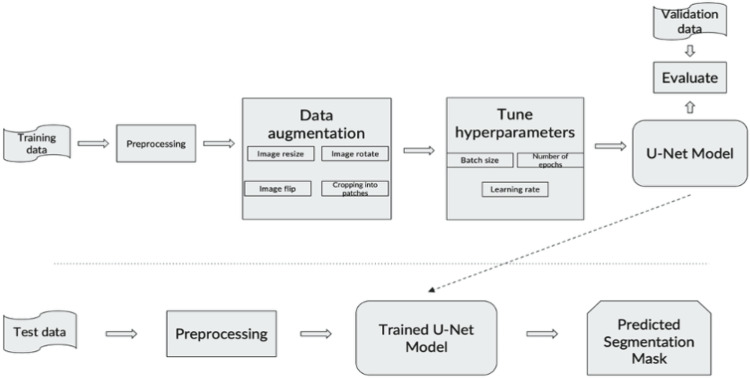
Workflow for model development and evaluation. Preprocessing and augmentation were applied to training data prior to model training and validation. Test data underwent preprocessing and were used to generate predicted segmentation masks. Figure created using Google Slides (Google LLC, Mountain View, CA, USA).

This retrospective feasibility study included 22 patients who underwent T2 MRI of the internal auditory canals at a single tertiary academic institution. Inclusion criteria consisted of availability of high-resolution axial CISS imaging suitable for segmentation and absence of prior skull base surgery or severe motion artifact. The overall study design and model development workflow are summarized in Figure 1. All imaging data were de-identified prior to analysis. The dataset was randomly divided into training (70%, n = 15) and validation (15%, n= four) subsets, with 15% (n = three) of cases reserved for model testing.

MRI acquisition

MRI scans were acquired between January 2015 and January 2025. All imaging used in the dataset was performed using a standardized institutional protocol on both a 3T Siemens MAGNETOM Vida scanner (Siemens Healthineers, Erlangen, Germany) and a 1.5T Siemens MAGNETOM Vida scanner (Siemens Healthineers), both operating on syngo MR XA60 with a 20-channel head and neck coil. High-resolution axial CISS imaging was acquired with typical protocol values, such as: repetition time (TR) 5.43 ms, echo time (TE) 2.42 ms, flip angle 62°, field of view 180 mm, acquisition matrix 512 × 512, in-plane resolution 0.35 × 0.35 mm, slice thickness 0.7 mm, and pixel bandwidth 425 Hz/pixel.

Manual segmentation

The facial nerve and vestibulocochlear nerve were manually segmented along the cisternal and intracanalicular segments on axial CISS images using dedicated medical image analysis software (3D Slicer (v5.4)) by a neurotology fellow. Segmentation was performed slice-by-slice to generate three-dimensional binary masks for each nerve. All segmentations were reviewed for anatomic accuracy prior to use as ground truth labels for model training, with final segmentations additionally reviewed by a senior neurotologist.

Image preprocessing

Digital imaging and communications in medicine (DICOM) images were imported and converted to volumetric arrays for analysis. All volumes were maintained in native image space. Given the consistent acquisition protocol and uniform spatial resolution across patients, inter-scan registration was not performed. Intensity normalization was applied prior to model training to standardize input data. Where required by model architecture, volumes were resampled to isotropic resolution and standardized input dimensions.

Model architecture and training

Deep learning-based segmentation was performed using three-dimensional convolutional neural network architectures derived from the U-Net framework. A 3D U-Net model served as the baseline architecture. In addition, three previously published U-Net-based variants implemented in the MONAI framework were evaluated, including Attention U-Net [11], Dynamic U-Net [12], and U-Net++ [13]. A modular training pipeline was developed to allow interchangeable model selection while preserving identical preprocessing steps, data augmentation strategies, loss functions, and training parameters across experiments. The dataset was divided into training (70%, n = 15), validation (15%, n = 3), and test (15%, n = 4) subsets. All models were trained to predict binary segmentation masks corresponding to manually labeled facial and vestibulocochlear nerves. Training was performed for 200 epochs using a combined Dice and cross-entropy loss function (DiceCE), with a learning rate of 0.0005 and a batch size of one. During training, patch-based sampling was employed to enhance dataset diversity and improve exposure to small anatomical structures. For each MRI volume, 16 randomly sampled patches of size 48 × 128 × 128 voxels were extracted per epoch using label-aware cropping. Data augmentation included random geometric transformations such as rotation and flipping. Model selection and hyperparameter optimization were performed using the validation set at five-epoch intervals.

Evaluation metrics

Segmentation performance was evaluated using the Dice similarity coefficient to quantify spatial overlap between predicted segmentation masks and manually generated ground truth labels. Dice scores were calculated on the held-out test dataset to assess model performance. No inferential statistical comparisons were performed given the exploratory nature and limited sample size of the study. In addition to quantitative evaluation, qualitative expert visual assessment was performed to compare anatomical plausibility and nerve delineation across model outputs.

## Results

Qualitative segmentation performance

**Figure 2:**
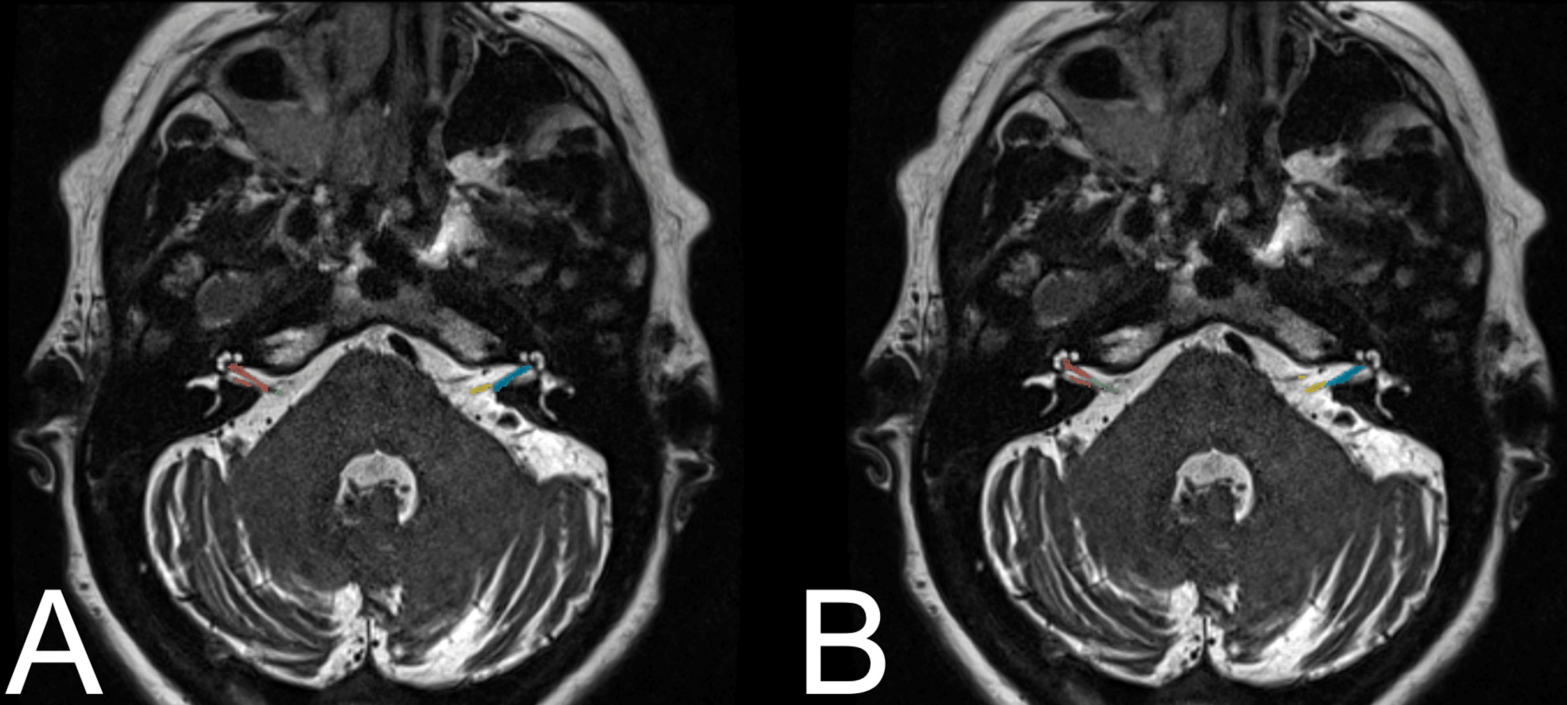
Ground truth and predicted segmentation of CN VII and CN VIII on axial CISS MRI. A: Ground truth. B: Prediction. Green = Right CN VII; Red = Right CN VIII; Yellow = Left CN VII; Blue = Left CN VIII Abbreviations: CN = cranial nerve, CISS = constructive interference in steady state

**Figure 3:**
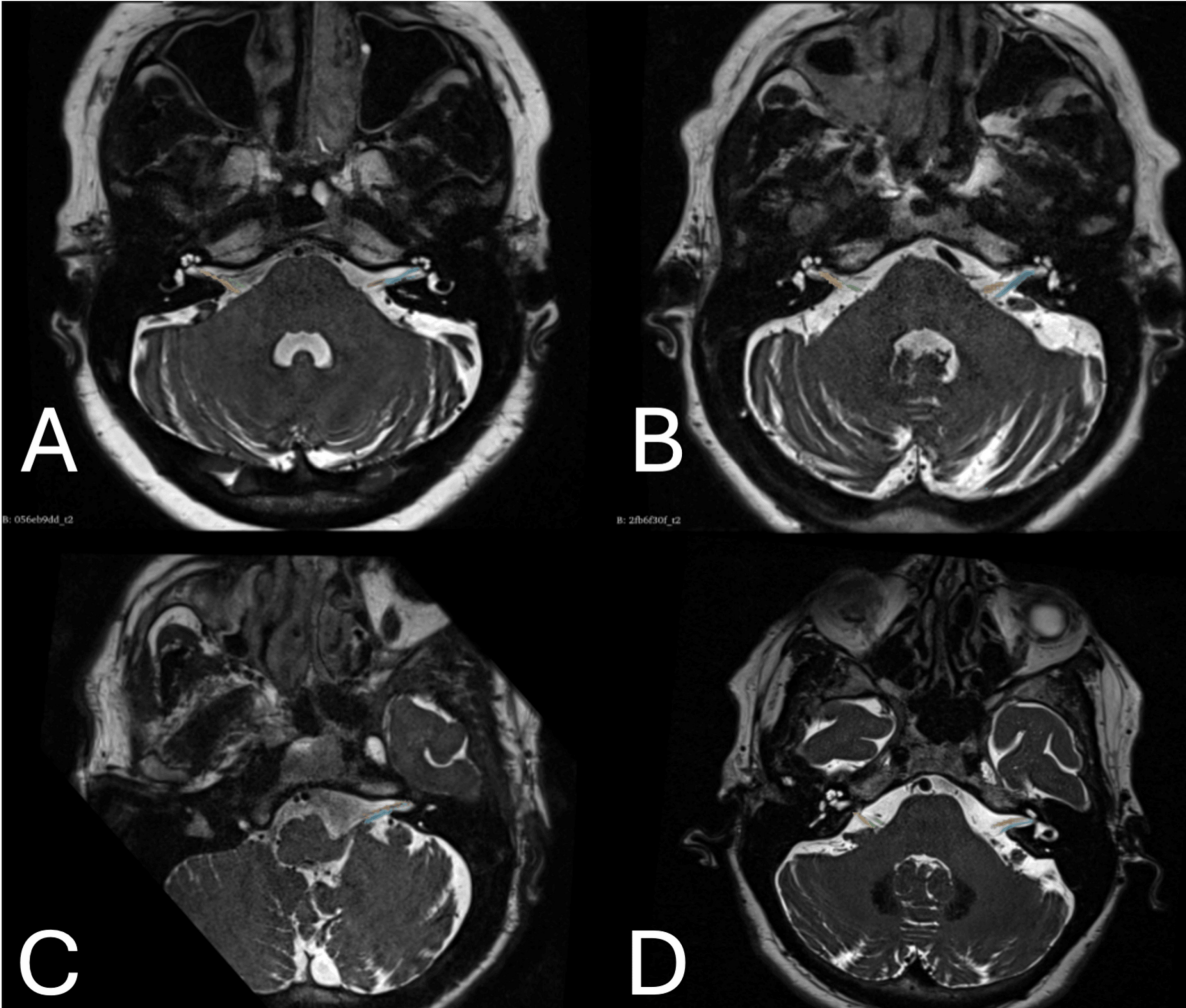
Predicted segmentation outputs for all test cases on axial CISS MRI. Abbreviation: CISS = constructive interference in steady state

Visual inspection demonstrated accurate bilateral delineation of CN VII and CN VIII, with continuous segmentation from the brainstem to the internal auditory canal fundus. Representative examples comparing manual ground truth and predicted segmentation are shown in Figure 2. Additional predicted segmentation outputs from all test cases are provided in Figure 3. Minor under-segmentation near the fundus was observed in some predictions, likely reflecting reduced MRI contrast at distal nerve segments.

Baseline model performance (3D U-Net)

For the baseline 3D U-Net model, the validation Dice similarity coefficient was 0.5609 and the test Dice similarity coefficient was 0.6398. Although numerically modest, these values are consistent with the known difficulty of segmenting thin neural structures with limited voxel representation. Nerve-specific test Dice scores for the baseline model were 0.5827 (right CN VII), 0.5925 (right CN VIII), 0.7300 (left CN VII), and 0.6539 (left CN VIII) (Table 1).

**Table 1:** Nerve-Specific and Overall Test Dice Scores by Model. Abbreviations: CN = cranial nerve; R = right; L = left *Failed to segment right-sided nerves, resulting in Dice scores of 0 for those structures

	U-Net	Dynamic U-Net	U-Net++	Attention U-Net
R CN VII	0.5827	0.5993	0.5807	0^*^
R CN VIII	0.5925	0.5701	0.6652	0^*^
L CN VII	0.7300	0.7456	0.7309	0.1910
L CN VIII	0.6539	0.6949	0.7096	0.5271
Overall test DICE	0.6398	0.6525	0.6716	0.1795

Training dynamics across model architectures

**Figure 4:**
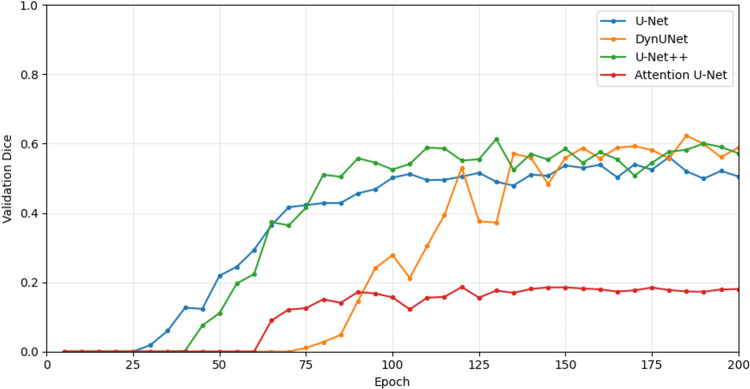
Validation Dice similarity coefficients for all model architectures (U-Net, Dynamic U-Net, U-Net++, and Attention U-Net) evaluated every five epochs.

Validation Dice scores improved progressively during training across all model architectures (Figure 4), with early rapid gains for U-Net and U-Net++ followed by convergence toward later epochs. U-Net++ and Dynamic U-Net demonstrated consistently higher validation Dice values compared to the baseline U-Net, while Attention U-Net showed limited performance.

Comparative model performance

**Figure 5:**
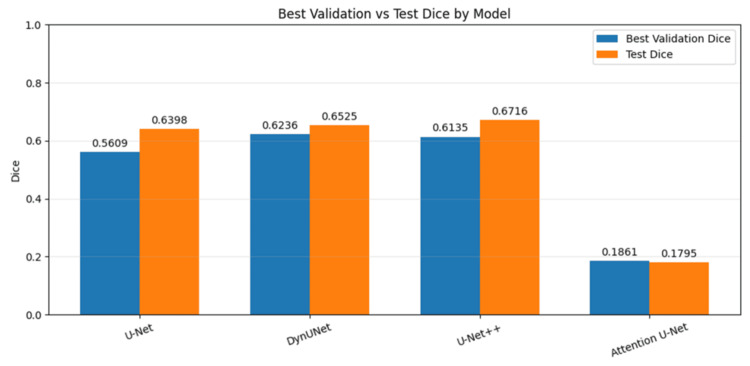
Best validation and test Dice similarity coefficient by model architecture.

Comparative evaluation of U-Net variants revealed architecture-dependent performance differences (Figure 5). All models were trained using identical datasets, preprocessing steps, and augmentation strategies. Attention U-Net demonstrated lower and less consistent performance, likely reflecting the challenges of extreme class imbalance and minimal voxel representation of cranial nerves. The baseline U-Net achieved moderate performance, while both Dynamic U-Net and U-Net++ demonstrated improved Dice scores. Nearing 200 training epochs, Dynamic U-Net achieved the highest validation Dice score (0.6236) within this dataset, while U-Net++ demonstrated relatively higher performance on the held-out test set, achieving a test Dice of 0.6716. For U-Net++, nerve-specific test Dice scores were 0.5807 (right CN VII), 0.6652 (right CN VIII), 0.7309 (left CN VII), and 0.7096 (left CN VIII) (Table 1). Given the limited size of the held-out test set, these performance estimates should be interpreted cautiously and primarily as descriptive indicators of model behavior rather than definitive comparative outcomes. Given the limited sample size and small test set, these differences should be interpreted as descriptive trends rather than definitive evidence of comparative model superiority.

## Discussion

Several prior studies have evaluated deep learning approaches for segmentation and automated identification of cranial nerves, including segmentation of the trigeminal nerve and surrounding vasculature [5], temporal bone structures on CT [6], and multimodal cranial nerve tract segmentation [7]. The present study extends these approaches to the individual facial and vestibulocochlear nerves within their cisternal and intracanalicular segments on high-resolution CISS MRI. Our findings demonstrate the technical feasibility of automated segmentation of CN VII and CN VIII using U-Net based architectures.

Although Dice similarity coefficients were modest, qualitative assessment demonstrated consistent anatomic overlap between predicted and manual segmentations (Figure 2). Lower Dice scores are expected when evaluating very small-caliber structures due to voxel-level sensitivity and limited foreground representation. Even minimal boundary discrepancies can disproportionately reduce Dice values while maintaining visually acceptable segmentation overlap. These findings align with prior reports showing decreased performance when segmenting fine or low-contrast neural structures compared with larger anatomical targets [6,7]. Importantly, this feasibility study was conducted in a limited dataset (n = 22), and the primary aim was proof-of-concept rather than performance optimization.

Previous deep learning studies have applied U-Net-based architectures to segment small neural and vascular structures with varying success. A mean Dice score of approximately 0.78 was achieved for the trigeminal nerve and adjacent vasculature in patients with trigeminal neuralgia [5]. Other work has shown lower Dice scores for fine or low-contrast anatomy, such as the facial nerve in temporal bone CT (0.703) compared with larger structures, such as the labyrinth (0.910), underscoring the increased difficulty of segmenting fine, low-contrast anatomy [6]. Similarly, segmentation of cranial nerve tracts remains challenging due to their slender morphology and low tissue contrast [7]. Taken together, these reports indicate that despite strong performance of U-Net architectures, segmentation accuracy typically declines for smaller caliber or low-contrast neural structures, which is consistent with the somewhat lower Dice scores observed in the present study. Consistent qualitative anatomic overlap was observed despite the limited dataset (n = 22), supporting the feasibility of automated segmentation in this setting.

The progressive increase and convergence of training and validation Dice scores suggest stable learning dynamics of the U-Net model. Similar training-validation behavior has been reported in other medical image segmentation studies using U-Net variants and nnU-Net, where training dynamics, such as gradual improvements in training and validation metrics, and architecture configuration appears to support stable learning [1-3,8]. Given that nnU-Net and related architectures are implemented in MONAI, future directions could leverage these frameworks to streamline experimentation and assess potential performance gains. Data augmentation strategies such as patch-based sampling and geometric transformations are discussed as common means to reduce overfitting and improve generalization for small or scarce targets [4]. In the context of small-structure segmentation, studies of fine neural or vascular anatomy (e.g., small caliber nerves) show that U-Net models can nonetheless achieve clinically useful overlap despite limited volumes of training data [9]. 

The comparative evaluation of U-Net variants (Figure 5) suggests that architectural selection may influence segmentation performance in small-caliber neural structures. While attention mechanisms have improved performance in tasks involving larger or higher-contrast targets, their suppression of weak signals likely limits utility in cranial nerve segmentation, where the foreground occupies a minimal proportion of voxels. The poor performance of the Attention U-Net is likely attributable to the extremely small voxel-wise representation of the cranial nerves, where attention mechanisms may suppress low-intensity but anatomically meaningful signals. In contrast, Dynamic U-Net adapts receptive field configuration and depth based on input resolution, which may better preserve spatial context for fine neural anatomy. Additionally, the nested skip connections of U-Net++ enable more effective multi-scale feature fusion and improved semantic alignment between encoder and decoder representations, resulting in more precise boundary delineation of small anatomical structures. This could explain why it generalized the best on unseen test data compared to other architectures. Our results empirically support the utility of MONAI-based models for rapid prototyping and benchmarking of segmentation models, even in limited samples, and suggest that Dynamic U-Net and U-Net++ models may be particularly well suited for small-structure segmentation tasks. However, the present study was not designed or powered for formal model comparison or performance ranking, and observed differences between architectures should be interpreted as exploratory and hypothesis-generating.

Several methodological limitations should be acknowledged. First, the study is not powered for inferential statistical comparison, and performance differences between model architectures should be interpreted as descriptive rather than confirmatory. Second, the test dataset was small and derived from the same single-institution cohort, which limits representation of anatomical variability and introduces potential selection bias, thereby constraining generalizability. Third, evaluation relied primarily on the Dice similarity coefficient, which has known limitations when applied to small-caliber structures such as cranial nerves. In this setting, small boundary discrepancies may disproportionately reduce Dice values, while conversely, spatial overlap metrics may overestimate clinically meaningful segmentation accuracy. These factors reinforce that the present findings should be interpreted as preliminary and hypothesis-generating within a feasibility framework.

Automated segmentation of the facial and vestibulocochlear nerves may ultimately support standardized nerve visualization, cochlear implant candidate assessment, and surgical planning in lateral skull base procedures. The present study establishes preliminary technical groundwork for these future investigations.

## Conclusions

Deep learning-based segmentation of the facial and vestibulocochlear nerves on high-resolution MRI is technically feasible within a limited retrospective dataset. Although Dice similarity coefficients were modest, consistent qualitative anatomic overlap with manual labels was observed. Model architecture may influence segmentation performance, with Dynamic U-Net and U-Net++ demonstrating relatively improved results within this limited dataset. These findings establish preliminary technical groundwork for future studies incorporating larger, multi-institutional cohorts and pathologic anatomy to further evaluate robustness and potential clinical applicability.
